# The Role and Therapeutic Targeting of CCR5 in Breast Cancer

**DOI:** 10.3390/cells12182237

**Published:** 2023-09-08

**Authors:** Rasha Hamid, Mustafa Alaziz, Amanpreet S. Mahal, Anthony W. Ashton, Niels Halama, Dirk Jaeger, Xuanmao Jiao, Richard G. Pestell

**Affiliations:** 1 Xavier University School of Medicine, Oranjestad, Arubaamanpreet.mahal@students.xusom.com (A.S.M.);; 2 Lightseed Inc., Wynnewood, PA 19096, USA; 3 Lankenau Institute for Medical Research Philadelphia, Wynnewood, PA 19096, USA; 4 Department of Medical Oncology, National Center for Tumor Diseases (NCT) Heidelberg, Heidelberg University Hospital, 69120 Heidelberg, Germany; niels.halama@nct-heidelberg.de (N.H.); dirk.jaeger@nct-heidelberg.de (D.J.); 5 Department of Translational Immunotherapy, German Cancer Research Center (DKFZ), 69120 Heidelberg, Germany; 6 Clinical Cooperation Unit Applied Tumor-Immunity, 69120 Heidelberg, Germany; 7 Pennsylvania Cancer and Regenerative Medicine Research Center, Baruch S. Blumberg Institute, Wynnewood, PA 19096, USA; 8 The Wistar Cancer Center, Philadelphia, PA 19107, USA

**Keywords:** CCR5, breast cancer, triple-negative breast cancer

## Abstract

The G-protein-coupled receptor C-C chemokine receptor 5 (CCR5) functions as a co-receptor for the entry of HIV into immune cells. CCR5 binds promiscuously to a diverse array of ligands initiating cell signaling that includes guided migration. Although well known to be expressed on immune cells, recent studies have shown the induction of CCR5 on the surface of breast cancer epithelial cells. The function of CCR5 on breast cancer epithelial cells includes the induction of aberrant cell survival signaling and tropism towards chemo attractants. As CCR5 is not expressed on normal epithelium, the receptor provides a potential useful target for therapy. Inhibitors of CCR5 (CCR5i), either small molecules (maraviroc, vicriviroc) or humanized monoclonal antibodies (leronlimab) have shown anti-tumor and anti-metastatic properties in preclinical studies. In early clinical studies, reviewed herein, CCR5i have shown promising results and evidence for effects on both the tumor and the anti-tumor immune response. Current clinical studies have therefore included combination therapy approaches with checkpoint inhibitors.

## 1. Introduction

Breast cancer represents 31% of estimated new cancer cases in the USA (first place in women) and is the second leading cause of cancer in women, with 15% of estimated deaths for 2023 [1]. Breast cancer staging determines therapeutic options and correlates with 5-year relative survival [1]. According to the National Cancer Institute, between 2015–2019, the age-adjusted death rate was 19.9 per 100,000 women per year [2].

The standard Nottingham combined histologic grade is the classic histopathological grading system that provides a significant prognostic tool to predict the outcome of a particular breast tumor [3]. In addition to the traditional histological grading, the molecular classification of breast cancer based on biomarker expression can provide further information to determine the biological behavior of the tumor and can help in deciding the treatment strategies [4,5]. Molecular classifications of breast cancer include luminal A, luminal B, HER2 (human epidermal growth factor receptor 2) enriched, and basal-like, which is also known as triple-negative breast cancer (TNBC) [6,7]. In general, compared with the basal-like and HER2-related subtypes, the luminal subtypes express estrogen receptor alpha (ERα) and have a better prognosis.

TNBC has a high risk of invasiveness, greater metastatic potential, and does not respond to hormonal or HER2-targeted therapy [6,8]. Patients with TNBC have a less favorable prognosis, with a 77.1% 5-year survival rate, compared to 84.8–94.4% for other types of breast cancers [2]. Based on SEER databases, the 5-year survival rate for local TNBC patients is 91.3%. If the tumor has spread locally or to nearby lymph nodes, the 5-year survival rate decreases to 65.8%, and distant metastases are associated with a 12.0% 5-year survival rate [2]. Therefore, the need for effective treatment of TNBC remains an urgent necessity. A study of 2245 human breast cancer samples showed increased expression of CCR5 and its ligand CCL5 in most basal and HER2 subtypes [9]. Over 95% of TNBC tumors expressed CCR5 [8,10]; therefore, increased CCR5 expression and elevated CCL5 levels predict a poor prognosis of breast cancer [10]. CCL5 levels correlate with advanced breast cancer stage in several studies [9]. CCR5 was shown to be an essential participant in breast cancer metastasis using CCR5i [9]. Furthermore, CCR5 contributed to tumor growth, drug resistance, tumor migration, and prognosis [10,11]. Reconstitution of CCR5 expression in CCR5-negative breast cancer cells imbued the cells with mobility and metastatic capability, induced DNA repair gene expression and activity, and promoted stemness, resulting in the ability to form new tumors in mice [11].

Although a matter of ongoing controversy, a mutation in CCR5 was reported to be associated with a lower risk of metastasis in breast cancer [12], and CCR5 expression in other cancers has been linked to recurrence risk and prognosis [13]. In postmenopausal women, the mutant allele CCR5-delta32 (CCR5del32), which has a negative effect on the function of the wildtype CCR5 and is increased in prevalence in the northern European population, is associated with favorable prognosis and less metastasis [12]. Recognizing the limited treatment options available for patients with TNBC, recent clinical studies have targeted CCR5. The role of CCR5 in the progression of breast cancer and the importance of blocking CCR5 in treating breast cancer are reviewed herein.

## 2. Normal Physiology of CCR5

**Figure 1:**
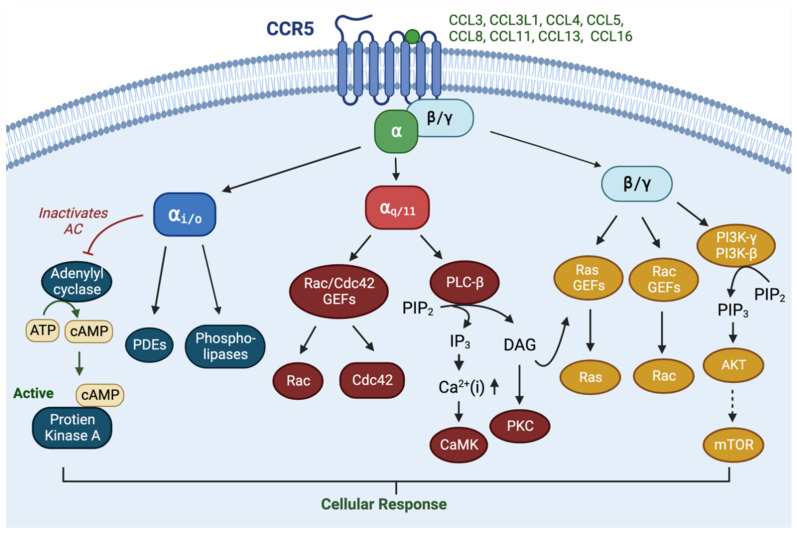
**Normal physiology of the CCR5-CCL5 axis.** CCR5 is shown as a transmembrane receptor linked to the activation Gαi/o, Gαq/11 and Gβγ and their downstream signal pathways.

CCR5 is a seven-transmembrane G-protein-coupled receptor (GPCR). GPCRs coupled to heterotrimeric G proteins consist of three subunits—Gα, Gβ, and Gγ. There are 16 Gα_s_ in mammalian cells, divided into four classes—Gα_i/o_, Gα_s_, Gα_12/13_, and Gα_q/11_ [14,15]. CCR5 predominantly couples to Gα_i/o_ and Gα_q/11_ [16], is expressed on dendritic cells, T cells, macrophages, eosinophils, microglia, and myeloid-derived suppressor cells [10,17] and is activated by diverse ligands (CCL3, CCL3L1, CCL4, CCL5, CCL8, CCL11, CCL13, and CCL16) [18,19] to promote chemotaxis and immune cell activation. CCL7 also binds to CCR5, but without a biological function, and can act as a natural CCR5 antagonist [18]. Upon ligand binding, CCR5 undergoes conformational changes which enable GTP loading to associated heterotrimeric G proteins and dissociates it to GTP-bound Gα and Gβγ [16] (Figure 1).

**Figure 2:**
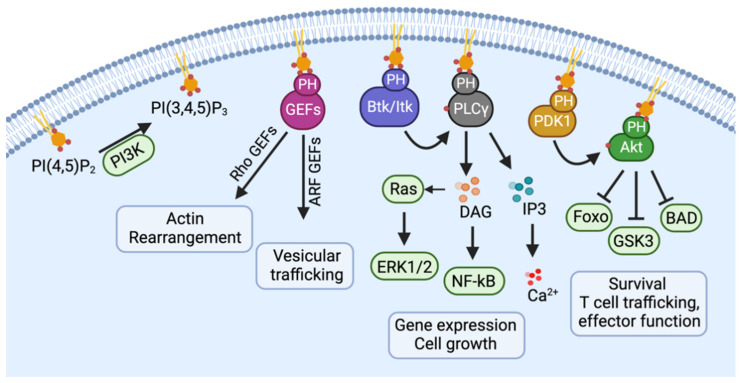
**The activation of PI3K results in the conversion of phosphatidylinositol-2 (PIP2) to phosphatidylinositol-3 (PIP3).** PIP3 will recruit a Pleckstrin Homology (PH) domain containing proteins, including Rho-GEFs, PDK1, and Akt, as well as Btk/Itk and PLCγ, to the plasma membrane for activation.

The main functions of Gα_i_ are inhibiting cAMP production, activating a variety of phospholipases and phosphodiesterases, and promoting the opening of several ion channels [20,21]. The Gα_q/11_ family activates Protein Kinase C (PKC) and elevates intracellular Ca^2+^ levels via the conversion of phosphatidylinositol 4,5-bisphosphate to Diacylglycerol (DAG) and inositol-1,4,5-trisphosphate [20,21]. Although DAG typically activates Ras-Guanine Nucleotide Exchange Factors (GEFs) [22], the Gα_q/11_ subunit noncanonically stimulates Rho GTPases via Rho GEFs [23,24]. Once dissociated from the Gα, Gβγ dimers can activate Phospho-Inositol-3-Kinases (PI3K), Phosphol-lipase-Cβ (PLCβ), adenylyl cyclase, and the small GTPases Rac, which are stimulated by GEFs [20]. The activation of PI3K results in the conversion of phosphatidylinositol-2 (PIP2) to phosphatidylinositol-3 (PIP3), which will recruit a Pleckstrin Homology (PH) domain containing proteins to the plasma membrane for activation [25] (Figure 2). These proteins include Rho-GEFs, Pyruvate Dehydrogenase Kinase-1 (PDK1) and Akt, as well as Btk/Itk and Phospholipase-Cγ (PLCγ). Rho-GEFs stimulation of small GTPases such as Rac result in actin rearrangement. Akt is phosphorylated by PDK1, thereby activating the Akt/mTOR (mammalian target of rapamycin) and mitogen-activated protein kinase (MAPK) signaling pathways, which contributes to cell survival and drug resistance. Although Gβγ can activate PLCs, generating IP3 and DAG, calcium elevation induced by CCR5 is mostly from Gα_q/11_ activation [16].

Although initially characterized in immune cells, CCR5 signaling in MDA-MB-231 breast cancer cells showed CCL5 and serum-induced calcium signaling [11]. Analysis of signaling pathways in breast cancer cells showed that CCR5 augmented ribosomal biogenesis, PI3k/Akt1 HIF1, and focal adhesion signaling [11]. Western blot analysis confirmed that CCR5 inhibition reduced Akt phosphorylation [11]. Single-cell sequencing comparing CCR5^+^ vs. CCR5^−^ breast cancer cells from within a heterogeneous population of breast cancer patients revealed dramatic enrichment of pathways governing ribosomal biogenesis [11].

Functional studies of CCR5 in breast cancer cells revealed similar attributes as immune cells promoting cellular chemotaxis, activation of immune mediators, and cellular proliferation [10,17]. The CCL5-mediated induction of cellular invasion was observed in diverse breast cancer cell types (MDA-MB-231, Hs587T, SUM-159, MCF-7, MCF10A-NeuT, MCF10A-Ras, and MCF10A-Src [11]). Understanding the function of CCR5 in physiological conditions helps predict plausible roles it may play in tumor development and metastasis progression.

## 3. Pathophysiology of CCR5 in Cancers

The pathological expression of CCR5 has been demonstrated in many different types of tumors, including breast cancer [9,11], prostate cancer [26], colorectal carcinoma, pancreatic cancer, melanoma, head and neck cancer, gastric cancer, esophageal cancer, Hodgkin lymphoma, and acute lymphocytic leukemia [27,28,29,30,31,32,33,34,35]; reviewed in [10,30]. Elevated levels of CCL5 in tissues or plasma predicts poor outcomes in breast cancer [36,37], cervical cancer [37], prostate cancer, ovarian cancer [38], gastric cancer [39,40], and pancreatic cancer. Elevated levels of CCL5 predict poor response to regorafenib in metastatic colorectal carcinoma [41]. CCL5 levels are high in patients with hot melanoma vs. cold melanoma and CCL5-high tumors express more effector immune cells (Th1, NK, CD8^+^ T cells) [42].

CCR5 abundance is induced in immortalized breast cells upon oncogenic transformation by v-Src, Ha-Ras, ErbB2, or c-Myc, or by DNA damage, or CCL5 stimulation [9]. CCR5 expression on breast cancer cells results in increased cancer cell motility, strengthened DNA damage repairing, and enhanced capacity of tumor cells to survive and resist chemotherapeutic regimens [11]. Hypoxia also activates CCR5 and CCL5 expression in breast cancer cells [43].

In an analysis of >2200 breast cancer patients, >95% of triple-negative breast cancer (TNBC) were CCR5^+^ [9]. More than half of all patients’ tumors were CCR5^+^ [9]. Higher cytoplasmic CCR5 staining predicted a poorer outcome [11]. CCR5 was also overexpressed in >90% of Her2+ BCa, and 30–40% of luminal breast cancers.

In addition to direct effects on the breast cancer epithelial cell, the CCL5-CCR5 axis may promote tumor progression by modulating the breast cancer tumor microenvironment [44]. In HER2^+^ murine breast cancer, after tumor regression, CCL5 remained elevated in the residual tumor, recruiting Tumor-Associated Macrophages (TAMs) that promoted tumor recurrence [45]. Malignant phyllodes breast tumor, which is derived from breast cancer periductal stromal cells, lacks effective therapy and is, therefore, another area of unmet need. The CCL5-CCR5 axis promotes tumor growth of phyllodes tumors via recruitment of TAMs [46].

CCR5 on the tumor surface may respond in an autocrine manner to ligands secreted by the cancer cell or in a paracrine manner to ligands secreted by the cells of the tumor microenvironment, including T cells, myeloid-derived stem cells (MDSCs), and macrophages. The binding of CCL5 to CCR5 on cancerous epithelial cells induces tumor growth via several signaling pathways, including the mammalian target of rapamycin (mTOR), the pathway activation of the Janus kinase signal transducer, and the activator of transcription (JAK-STAT) pathway [9,11].

## 4. The Role of CCR5 in Breast Cancer Cell Metabolism

The growth of a tumor depends upon its ability to metabolize energy. The primary energy source in tumor cells is aerobic glycolysis via the Warburg effect [47,48,49]. Normally, cells produce energy through oxidative phosphorylation in the mitochondria; however, cancer cells produce energy via aerobic glycolysis in the cytosol, even in the presence of oxygen, resulting in lactate production and secretion into the local tumor microenvironment [48,50,51]. CCR5 promotes glucose uptake in breast cancer cells to maintain energy supply during tumor growth [50,52]. As a result of continuous cancer cell growth, there is a massive increase in energy expenditure, leading to an increase in oxygen consumption and hypoxia. Glucose transport protein (GLUT) mediates glucose uptake across the cell membrane [50]. There are many GLUT isoforms; however, in cancer cells, only GLUT 1, GLUT 3, and GLUT 12 mediate glucose uptake [53]. The CCL5-CCR5 axis and GLUT 1 both use the Akt (protein kinase B) pathway. CCR5 increases the cell surface GLUT-1 expression (but not other GLUT isoforms), which mediates glucose uptake and energy supply to cancerous cells [50].

## 5. The Role of CCR5 in Tumor Migration, Circulating Tumor Cells, and Tumor Metastasis

**Figure 3:**
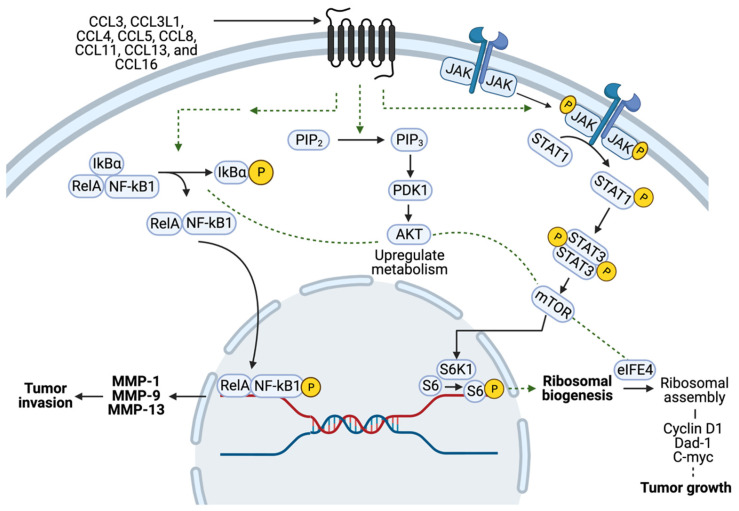
**Schematic representation of CCR5 signaling in breast cancer cells.** The activation of CCR5 by ligand induces downstream signaling pathways impacting ribosomal biogenesis in breast cancer and induces several signaling pathways, including NF-κB, Akt, and STATs, contributing to protein synthesis, cellular growth, and migration.

CCR5 has been implicated in the progression and metastatic spread of different cancers, including breast cancer, prostate cancer, glioblastoma, osteosarcoma, and oral cancer [9,26,54,55,56,57,58], reviewed in [59]. CCR5-positive cells may be more migratory in nature and thus more likely to become metastatic [55,60]. Elevated CCR5 signaling is associated with highly invasive breast cancer subtypes [10,50,61,62]. The upregulation of CCR5 signaling in breast cancer is positively correlated with axillary lymph node metastasis, consistent with a model in which CCR5 correlates with the spread of more aggressive disease [12,62]. Tumor migration induced by CCR5 involves multiple distinct pathways. Firstly, the binding of CCL5 to CCR5 activates PI3K/Akt and NF-κB (nuclear factor kappa-light-chain-enhancer of activated B cells) (Figure 3), resulting in the activation of αvβ3 integrin, which mediates cell migration [52]. Secondly, tumor metastasis requests that cancer cells travel through the blood and lymph system. Circulating tumor cells are the tumor cells in the blood of patients, which can be measured with several cancer types [63].

CCR5 is expressed on circulating tumor cells (CTC) of breast cancer patients [64,65]. The finding that CCR5 is expressed on CTC is not necessarily expected, as the gene expression of CTC varies substantially from the primary tumor, both in breast cancer [66,67] and other cancer types [68]. The change in gene expression is necessary to enable the process of invasion, intravasation, and survival, since cells within circulation are often exposed to extreme turbulence and shearing forces. The treatment of patients with breast cancer metastasis using a CCR5 monoclonal antibody, leronlimab, was associated with a reduction in CTC in patients [69]. CCR5 signaling is not high in primary breast cancer tumor sites, but is upregulated in secondary sites of metastasis, which suggests the CCR5-CCL5 axis may play a role in upregulating circulating tumor cells (CTC), and thereby contribute to a poor clinical outcome [55,64,70,71].

Thirdly, tumors recruit tumor-associated macrophages (TAMs), which provide heterotypic signals to promote tumor cell intravasation, extravasation, and migration [10,52,72].

## 6. Involvement of CCR5 in Breast Tumor Angiogenesis

A tumor can stimulate neovascularization through cytokines or via hypoxia-mediated responses. A key mediator of tumor angiogenesis is the hypoxia-responsive protein Vascular Endothelial Growth Factor (VEGF) [73,74]. CCR5 activation triggers Protein Kinase C δ (PKCδ), followed by initiation of c-Src Kinase (Proto-oncogene tyrosine-protein kinase Src) and hypoxia-inducible factor-1 (HIF-1) expression, which results in stimulation of VEGF [74]. CCR5 antagonist resulted in less vasculature, and impaired tumor growth [75]. Inhibition of CCL5/CCR5 signaling impairs endothelial cell migration, associated with a decrease in activation of the mTOR/Akt pathway [75]. Using CCR5 knockout, elegant reconstitution experiments in a murine model of EO771 breast cancer showed CCR5 expressing endothelial cells in the breast tumor microenvironment contributed to ongoing breast tumor growth [75]. Furthermore, CCL5, from endothelial cells, acts in a paracrine fashion on triple-negative breast cancer cell lines (MDA-MB-231, Hs578t) to enhance their migration, invasion, and metastasis [76]. Overall, the CCR5-CCL5 axis induces a proangiogenic environment, promoting endothelial cell migration and neovascularization that provides the necessary nutrients and oxygen needed for tumor growth.

## 7. The Role of the CCR5-CCL5 Axis in Immune Evasion

A variety of studies have shown a role for CCR5-CCL5 in promoting tumor immune evasion and that inhibiting this axis promotes the anti-tumor immune response. Tregs are immunosuppressive and Treg cell recruitment has been shown to depend on CCL5 in tumors including breast cancer [77,78,79]. CCL5 expression has also been correlated with breast cancer poor prognosis [78], lymph node metastasis [78], residual tumor size, and tumor infiltration of lymphocytes after neoadjuvant chemotherapy [80]. In an inducible mammary-epithelial-cell targeted Her2 model of breast cancer, a tumor microenvironment inflammatory program driven by TNFα/NFκB signaling promoted immune cell infiltration via CCL5 [45]. CCL5 expression was also elevated in human residual breast tumors following treatment. CCL5 promoted breast cancer recurrence through recruitment of macrophages into the residual tumors [45], and a high TAM number has been associated with poor prognosis in breast cancer [81]. Breast cancer patients with high CCL5 expression had worse disease-free survival and breast cancer-specific survival [78]. CCL5 is thought to aid tumor cells’ escape the immune system, which is essential in tumor survival and progression in breast and other cancers [82,83]. Serum CCL5 levels in BCa patients correlated with lymph node metastasis [84] and analysis of Tregs, which promote tumor metastasis, showed CCR5^+^/CD4^+^ and Treg/CCR5^+^ cell ratios were significantly increased in the lymph nodes of the breast cancer metastasis group [78]. Although most studies have shown that high CCL5 correlates with poor outcomes in breast cancer [80], correlative studies have also shown a positive association between CCL5 and tumor-infiltrating lymphocytes (TILs) in TNBC. The functional relationship of CCL5 to the TILs in TNBC was not tested [80], and TNBC has since been shown to consist of several immune subtypes (discussed below).

**Figure 4:**
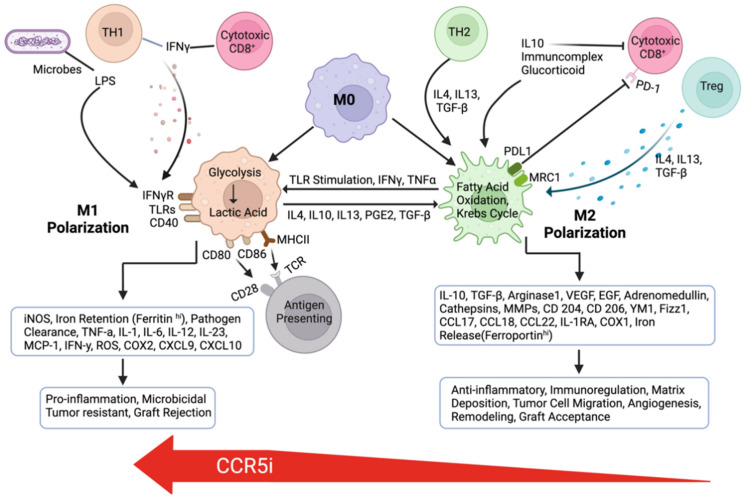
Formation of M1 and M2 macrophages in the tumor microenvironment. CCR5 inhibitor maraviroc promoted enrichment of M1 from M2 macrophages.

CCL5 may be breast-tumor-derived [36,37] or hematopoietically-derived [85], which promotes mammary tumor (4T1) progression via generating myeloid-derived suppressor cells (MDSCs) in the bone marrow [85]. CCL5 recruits monocytes from the circulation and reprograms them to become the M2 (alternatively activated macrophage) rather than M1 (classically activated macrophage) subtype [82,83]. Subsequently, M2 plays an essential role in the development of immune tolerance (Figure 4). M2-TAM secretes immunosuppressive cytokines, including transforming growth factor β (TGF-β) and interleukin-10 (IL-10). These cytokines enhance the effect of the immunosuppressive T regulatory cells (Tregs), which control macrophages, T cells, B cells, dendritic cells, and natural killer cells. M2 cells express programmed-death-ligand 1 (PD-L1), which can further aid in immune evasion. PD-L1 works as a break for the progression of the immune response by binding to the PD-1 receptor on lymphocytes and inhibiting downstream signaling cascades, which leads to the inhibition of CD8^+^ cytotoxic T cells, rendering them anergic, causing a further decrease in cell-mediated killing [86].

A recent study found that a higher number of CCR5-positive breast-cancer-associated macrophage-like cells in inflammatory breast cancer patients was correlated with poor prognosis [65]. Further analyses revealed a central role of the chemokine receptors CCR1 and CCR5 and their ligands as an immunological node induced by tumor-derived factors. Activation of this pathway is essential for the differentiation of myeloid-derived suppressor cells (MDSCs) and protumoral macrophages. Thereby, CCR5 and ligands mediate differentiation of tumor-associated myeloid cells and cancer-induced myelopoiesis [87].

## 8. The Role of CCR5 in Breast Cancer Stem Cell Expansion and Chemo-/Radio-Therapy Resistance

Cancer stem cells (CSCs) can self-renew and give rise to tumors with distinct cancer cell lineages [88,89,90]. CSCs can thereby contribute to tumor heterogeneity [48] and altered tumor metabolism [91]. CSCs participate in promoting metastasis and therapy resistance [88,89]. CSCs can be characterized by tumor sphere formation, cell surface markers (EpCAM^+^CD44^+^CD24^−^), and high activity of aldehyde dehydrogenase 1 [92]. Cancer cells’ growth as spheres with specific culture conditions predicts the ability to initiate tumorigenesis and the capacity to metastasize [93]. Tumor sphere formation thus reflects properties of CSCs [89,94,95]. Lineage ^−^CD44^+^CD24^−^ tumor cells isolated from breast cancer patients are enriched for cancer stem cells. Unlike ^−^CD44^+^CD24^−^ cells, which readily gave rise to tumors in mice, a thousandfold more cells with different immune phenotypes did not give rise to tumors [89]. Our laboratory found that a small population of basal breast cancer cells are CCR5 positive. Compared with CCR5^−^ breast cancer cells, the CCR5^+^ cells can form more mammospheres and are enriched with EpCAM^+^CD44^+^CD24^+^ cells [11].

When the same number of CCR5^−^ and CCR5^+^ breast cancer cells were implanted in mice, the tumors formed by CCR5^+^ cells were ~770-fold larger than those formed with CCR5^−^ cells [11]. The epithelial–mesenchymal transition (EMT) participates in the process of breast cancer cell transition into CSCs [96]. Transforming growth factor β (TGFβ) induction of EMT increases cancer cell invasiveness and promotes tumor metastasis [97,98,99]. TGFβ induces EMT though both Smad-dependent and -independent pathways [100]. The Smad-independent pathway involves the activation of PI3K/Akt/mTOR signaling [100,101]. CCR5 is upstream of PI3K/Akt/mTOR. The activation of CCR5 can enhance PI3K/Akt/mTOR activity [102], potentially contributing to increased EMT and CSC formation.

CCR5 conveyed stemness in several assays of breast cancer stem cells, enhancing mammosphere formation, and showing enrichment for tumor-initiating cells [11]. Other mediators of cancer stemness [91] intersect with CCR5/CCL5 signaling. Notch induced CCL5 secretion and CCR5 signaling in tumor-associated macrophages [44]. Cancer-cell-derived-lactate increased the secretion of CCL5 through Notch signaling in tumor-associated macrophages, and CCL5 in turn induced EMT and aerobic glycolysis in breast cancer cells. Notch signaling was reported to induce M1 macrophages [103] and inhibit tumor growth via anti-tumor immune responses. Notch1 regulates cell proliferation and migration through CCR5 in T cell acute lymphoblastic leukemia [104]. CCR5 antagonists decreased Notch signaling in B cells and in the brain in a murine model of multiple sclerosis [105].

Although primarily governing organismal size and functioning as a tumor suppressor pathway, the Hippo pathway participates in stem cell function. The transcriptional effectors YAP, TAZ, and YKI reside downstream of Hippo [95]. YAP regulates CTGF, Gli2, and other genes. Hippo intersects both the Wnt and Notch pathways, which in turn govern stem cell function [95]. YAP1 expression was positively correlated with Notch1 in breast cancer [106]. Breast tumors classified as poorly differentiated/high grade by histopathological criteria display elevated TAZ/YAP activity [107]. TAZ, a transducer of the Hippo pathway, is required to sustain self-renewal and tumor-initiation capacities in breast CSCs [107]. YAP1 expression was positively correlated with Notch1 in breast cancer [106], and CCL5/CCR5 activated YAP in tumor-associated macrophages [108]. CCL5 educated macrophages toward TAMs, which reciprocally enhanced clear cell renal cell carcinoma progression via CCL5/CCR5 and activated STAT3/SOX17^low^/YAP [108]. Collectively, these studies suggest important mechanisms by which CCR5/CCL5 may augment cancer stem cell expansion and the TME to induce a pro-tumorigenic environment.

CCR5 induces resistance to chemotherapy and radiotherapy through the induction of gene expression and function that govern DNA repair and DNA damage sensing. The breast cancer cells that survived from doxorubicin treatment were enriched with CCR5-positive cells compared with untreated cells [11]. CCR5 promotes stem-cell-like properties and enhances DNA repair [11]. In addition, resistance to tamoxifen is acquired through CCR5-CCL5 inhibition of apoptosis. CCL5 release promotes constitutive Signal Transducer and Activator of Transcription 3 (STAT3) phosphorylation. Subsequently, STAT3 phosphorylation promotes further CCL5 secretion and upregulation of BCL-2 (B-cell lymphoma 2) apoptosis inhibitors [109]. Resistance to trastuzumab is obtained by the interaction of CCL5-CCR5, leading to Extracellular Signal-regulated Kinase (ERK) phosphorylation [72], which leads to the stimulation of antiapoptotic cascades [102]. Taken together, these CCR5-CCL5 interactions contribute to CSC formation and therapy resistance in breast cancer patients.

## 9. CCR5 Inhibitors for Treating Breast Cancer

Preclinical studies have demonstrated the effectiveness of CCR5 inhibitors to reduce tumor growth and reduce the metastatic burden in mice [9]. CCR5 inhibition by maraviroc and vicriviroc blocked migration, invasion, and metastasis in immune-deficient mice. A dose of CCR5 inhibitor bioequivalent to the dose used in HIV patients, when used in mouse models, blocked breast cancer cell homing to the lungs [9]. Subsequent studies showed maraviroc reduced prostate cancer metastasis in immune-competent mice [26]. Leronlimab, a humanized IgG4 monoclonal antibody to CCR5, also showed promising preclinical efficacy, both reducing established metastasis and preventing the induction of human breast cancer metastasis in mice [58]. Leronlimab also inhibited CCR5 ligand-induced calcium signaling and cellular invasion in cultured human breast cancer cells [58].

Several clinical trials currently deploy CCR5 inhibitors for the treatment of refractory cancers (Table 1). The safety and efficacy of adding the CCR5 inhibitor maraviroc to pembrolizumab in refractory mismatch-repair-proficient colorectal cancer (MMRp CRC) was assessed in a phase I trial PICCASSO study. Analysis of the PICCASSO study [110] involving twenty patients with refractory colon cancer, who received pembrolizumab and maraviroc (core period, eight cycles), followed by pembrolizumab monotherapy, indicated feasibility and promising secondary endpoint responses. It needs to be mentioned that almost 75% of the patients had an unfavorable genomic profile (RAS mutations, BRAF mutations), also all had received the standard of care treatment, resulting in a very limited expected survival prognosis. In this heavily pretreated population, overall survival was higher than expected. The primary endpoint, the feasibility rate, was met (~95%). Secondary endpoints included safety/toxicity, overall response rate (ORR) (5.3%), progression-free survival (PFS) (2.10 months), and overall survival (OS) (9.83 months). The overall survival exceeded that of the historical control for this highly pretreated group. The control rate of disease was >70%.

A study of leronlimab with carboplatin (NCT 03838367) was initiated in patients with metastatic triple-negative breast cancer (TNBC). The study assessed safety and tolerability. As a phase 1b/2 study, the intent was to determine the maximum tolerated dose in order to define the phase 2 recommended dose (RP2D). The study received a fast-track designation in 2019 and showed promising results. CCR5 positivity was an entry criterion. Immunohistochemistry for CCR5 was defined as >10% CCR5 staining in breast tumor cells (primary or metastatic). CCR5+ cells also included CCR5+ tumor-infiltrating leukocytes (TIL). Patients were treated with a fixed dose of carboplatin (AUC 5 on day 1 with a 21-day dose-limiting toxicity window). To this regimen was added weekly subcutaneous leronlimab. Three dose levels of leronlimab were given using a 3 + 3 dose escalation regimen. Ten patients were enrolled at 3 dose levels, increasing from 350, to 525, and the highest dose of 700 mg. Eight of the previously unresponsive ten patients showed a response. Six out of ten patients achieved stable disease. The recommended dose of leronlimab for the future phase 2 study will be weekly leronlimab 700 mg and thrice-weekly carboplatin AUC5. The authors concluded that leronlimab, combined with carboplatin, was well tolerated at each dose level and that leronlimab showed early evidence of anti-tumor activity in CCR5^+^ metastatic triple-negative breast cancer patients [111]. In that regard, two out of ten patients achieved a confirmed partial response. Six of the ten patients achieved stable disease [111].

A related basket study of leronlimab (PRO 140) was used to treat patients with CCR5^+^ staining solid tumors that were either locally advanced or metastatic (NCT04504942). Pooled data (N = 19) was stated as showing >75% of patients showed improved median progression-free survival (mPFS) (6.1 months (95%CI 2.3–7.5)) and median overall survival (mOS) 12+ mos (95%CI 5.5–12+). The study was also said to show reduced circulating tumor-associated cells (TACs) in 75% (N = 21/28) of patients, which is thought to be a strong predictor of improved survival [112].

## 10. Triple-Negative Breast Cancer Subtypes and CCR5 Inhibitor Therapy

The use of CCR5 inhibitors in combination therapy for TNBC needs to consider evidence that distinct immunological subtypes of TNBC are now described and checkpoint inhibitors show promise in this patient population. TNBC includes both “hot” and “cold” tumors with immunological heterogeneity, and is recognized in both Caucasian [113] and Asian populations [114]. “Hot tumors” have significant T-cell infiltration (CD8^+^ cytotoxic T cells) and antigen-presenting cells such as dendritic cells are associated with better ICI (immune checkpoint inhibitor) efficacy. “Cold tumors” include impaired T-cell priming (reduced CD8^+^ T cells), increased numbers of suppressor cells (Tregs and myeloid-derived suppressor cells (MDSCs)), and deficient T-cell homing to tumor beds.

Four transcriptome-based subtypes of TNBC have been proposed: (1) The luminal androgen receptor (LAR) (23%) reflects signaling by the androgen receptor. (2) The immunomodulatory (IM) subtype (24%) is characterized by the activation of gene expression for immune pathways and cytokine signaling. In the immunomodulatory subtype, the immune recognition pathway is induced, consistent with a model in which this TNBC subtype may recruit immune-suppressive cells or activate immune checkpoints to escape the immune anti-tumor response. In patients with the TNBC IM, the adaptive immune response and interferon-gamma signaling gene expression are prominent [114]; these activated pathways coincide with the pathways that were most activated by GSEA analysis for CCCR5^+^ vs. CCR5^−^ breast tumors [11]. (3) In the basal-like immune-suppressed (BLIS) (39%) subtype, cell cycling, and DNA repair gene expression are induced, whereas immune response genes are suppressed. In the (4) mesenchymal-like (MES) (15%) genotype, there is induction of stem cell pathways, activated immune pathways, and enrichment of tumor-infiltrating lymphocytes (TILs) (stromal and intratumoral).

Other categories of the TME for breast cancer have been described. Analysis of ~10,000 cancers identified six immunogenomic subtypes, common to 33 cancer types. These subtypes are different from the traditional genomic and non-coding cancer classifications [115]. Breast cancer is enriched for C1 and C2, with a lesser amount of C3. In both C2 and C3 subtypes, an increased expression of IFNG and CCL5 correlated with increased NK cells together with CD4 and CD8 T cells. The C2, or IFN-g-dominant subtype, showed a relative increase in M1 vs. M2 macrophages, and an enrichment for TCR diversity. The C3, or “inflammatory”, subtype showed less chromosomal instability, with low levels of aneuploidy and somatic copy number alteration, and lower cell proliferation but elevated Th17 and Th1 gene expression. Although CCR5/CCL5 is associated with “hot tumors”, the functional significance of this axis, discussed below, appears to be immunosuppressive, making CCR5^+^ tumors potentially a target to potentiate checkpoint inhibition therapies.

## 11. Potential Role for CCR5 Inhibitors in Augmenting the Therapeutic Response to Current Breast Cancer Therapies

CCR5 inhibitors are administered (concurrently or sequentially) with DNA-damaging agents used to treat cancer, such as alkylating agents, intercalating agents, and polymerase inhibitors. Combined with existing chemotherapy, CCR5-blocking agents may provide several mechanisms to improve efficacy and reduce the dose of chemotherapy [11,58,116].

As noted above, an analysis of >2200 breast cancer patients showed that several different breast cancer subtypes determined by gene expression overexpress CCR5, including triple-negative breast cancer (TNBC), >90% of Her2+ BCa, and 30–40% of luminal breast cancer [9]. CDKi (palbociclib) is used for ERα^+^ Her2^−^ BCa [117]. In Her2+ BCa, Traztuzamab resistance of Her2+ breast cancer was linked to hyperactivation of CCL5/CCR5 signaling [118], and shown to be attenuated by maraviroc. Recent studies have shown that CDKi affects immune surveillance (please see our recent review, [117]). We hypothesize that CCR5i may either enhance or reduce the cell killing of CDK inhibitors, depending upon the tumor subtype and the tumor microenvironment [119]. We previously showed that cyclin D1 induces TILs and CCR5 ligand abundance [120]. Ras-MAPK-cyclin D1 pathway activation promotes immune evasion in TNBC [121]; however, CDKi has been shown to increase the anti-tumor immune response [122,123]. CDK4/6i increased anti-tumor immunity [122,124] and expression of CCL5 and PD-L1 [125]. As CCR5 binds with high affinity to several ligands that are induced by current BCa therapies (CCL5, CCL3 (MIP-1a), and CCL4 (MIP-1b), CCR5i may augment anti-tumor immune responses.

Radiation therapy is recommended for women treated with breast cancer conserving surgery. Radiation is also recommended for women with node-positive disease after mastectomy or with a tumor >5 cm. Radiation induces an abscopal effect. Low-dose radiation therapy induces a similar response to the effect seen with CCR5i (T cell and myeloid infiltration in tumors, and enrichment of anti-tumoral M1-like tumor-associated macrophages [126,127,128]. CCR5i enhances the DNA damage response of γ-radiation [11], raising the possibility that CCR5i may enhance the efficacy of radiation therapy for breast cancer patients.

Cytotoxic chemotherapy is recommended for patients with HER2 positivity (HER2^+^), HR-negative (HR^−^) status, or positive lymph nodes. In patients with ER-negative breast cancer receiving chemotherapy, patients with increased CCR5 populations showed a strong trend toward reduced metastasis-free and relapse-free survival compared with the patient population showing reduced CCR5 expression in their tumors [9]. Chemotherapy enhanced CD73 expression (mesenchymal stem cell, MSC) and PDL-1, which mediate cytotoxic T lymphocyte evasion [129]. CCL5 siRNAs reduced MSC-induced BCa metastasis [130]. Although chemotherapy may induce a pro-tumorigenic immune response [131], chemotherapeutics also activate the STING (STimulator of INterferon Genes) pathway [132], which induces CCL5 and may thereby induce PD-L1 expression and a tumor immunosuppressive environment [133]. In this circumstance, CCR5i would be predicted to reduce CCL5 action and mitigate the induction of the immunosuppressive environment.

## 12. Checkpoint Inhibitors

Several lines of evidence suggest CCR5 inhibition may augment immune checkpoint inhibitors or cellular immunotherapy (ICI) responses. CCR5 is found in “hot tumors” but appears to play a role in suppressing the anti-tumor immune response. Evidence includes the findings that: first, CCR5 promotes a tumor-suppressive tumor microenvironment. CCR5 pathway activation is necessary for the induction of protumoral macrophages and the differentiation of MDSCs [87]. CCR5 silencing of myeloid and myeloid precursors cells was sufficient to restrain tumor progression in vivo. The anti-tumor effect of CCR5 silencing correlated with the conversion of polymorphonuclear myeloid-derived suppressor cells into anti-tumor neutrophils. CCL3 in hematopoietic stem and precursor cells (HSPCs) activated CCR5 to convert HSPCs into MDSCs. Second, M2 macrophages, inflammatory monocytes, and myeloid cells such as myeloid-derived suppressor cells (MDSCs) drive tumor immune evasion. CCR5 is associated with MDSC accumulation, in melanoma [134,135]. Third, inhibiting CCR5 reduced the tumor-infiltrating MDSCs, and improved the survival rate in preclinical breast cancer and melanoma models [9,85,134].

The role of different cytokines in PDL1 inhibitor therapy may be more complex. Checkpoint-inhibitor-unresponsive melanomas lacked CCL4 (a ligand for CCR8—but also for CCR5 [136]). Restoration of CCL4 ligands restored responses to checkpoint inhibitors [137]. Intravenous administration of CBD-CCL4 (collagen-binding domain (CBD) of von Willebrand factor) increases tumor localization of CCL4 and thereby recruits CD8^+^ T cells and CD103+ DCs. The impact of CBD-CCL4 was to thereby improve the anti-tumor effect of checkpoint inhibitor therapy in melanoma and a breast cancer model (EMT6) including poor responders to CPI [137].

The composition of immune cell infiltration into the tumor influences the response to immune checkpoint inhibitor therapy. PD-L1 is not always a good predictor of the immune benefits of CPI [138]. The Keynote-522 trial (phase III) showed that the benefit of pembrolizumab occurred independently of PDL-1 status. In this regard, the pathological complete response rate among patients in the pembrolizumab arm was higher than that of the patients without anti-PD-1 treatment, independently of PDL-1 status [138]. Furthermore, checkpoint inhibitor therapy’s (atezolizumab and durvalumab) benefit for TNBC was independent of PDL-1 status in both the IMpassion031 and GeparNuevo studies [139,140].

Evidence suggesting CCR5 inhibition may augment immune checkpoint inhibitors or cellular immunotherapy (ICI) responses includes, firstly, evidence that CCR5 contributes to tumor progression by facilitating the recruitment of myeloid-derived suppressor cells and regulatory T cells to induce an immune-suppressive tumor microenvironment [141].

The second line of evidence that CCR5 inhibition may augment immune checkpoint inhibitors or cellular immunotherapy (ICI) responses includes animal model studies of gastric cancer in which an anti-CCR5 antibody decreased the number of tumor M-MDSCs and G-MDSCs. Furthermore, the combination of the anti-CCR5 antibody with an anti-PD-1 antibody treatment decreased the tumor burden in mice, correlating with increased tumor infiltration of CD4^+^ and CD8^+^ T cells [135].

Thirdly, in human colon cancer clinical trials, findings are consistent with CCR5 inhibitors reverting cold tumors [27,110]. Support comes from CCR5 inhibitor use in human colon cancer clinical trials. PD-1/PD-L1 inhibitors (PD-1 monotherapy or combined PD-1 and CTLA-4 blockade) do not show activity in colorectal cancers that are mismatch repair proficient (MMRp). However, when MMRp colorectal cancer was treated with maraviroc anti-tumoral M1, macrophages were activated, which is associated with the induction of pro-migratory chemokines and cytokines, increased T cell agglomeration, and improved median overall survival when compared with historic data (9.83 months). A combination treatment with two checkpoint inhibitors and CCR5 inhibition is currently ongoing, further enhancing synergistic activation of the adaptive arm of the immune system and the innate immunity [clinicaltrials.gov NCT04721301].

Collectively, therefore, CCR5-targeted therapy will be an effective treatment for patients with cancers that express high levels of CCR5/CCL5 and high levels of circulating tumor cells and/or metastases, and may be well positioned to augment the efficacy of current therapies.

## 13. Conclusions

CCR5 plays a pivotal role when expressed in breast cancers in facilitating tumor progression and metastasis in multiple ways. CCR5 stimulates tumor angiogenesis and induces tumor-cell-forming circulating tumor cells, which allow the tumor cells to spread to a distant site. CCR5 mediates tumor cell glucose uptake, which aids in tumor growth. The CCR5-CCL5 axis allows cancer cells to escape the immune system through the reprogramming of TAMs to become M2 macrophages and enhances the expression of PD-L1. CCR5 promotes resistance to chemotherapy through the stimulation of the DNA repair machinery and enhancement of anti-apoptotic mechanisms. CCR5 antagonists such as leronlimab, maraviroc, and vicriviroc have been shown to reduce the impact of CCR5 on tumor progression and halt the metastatic process. Data from clinical trials suggest that CCR5 blocking agents may be promising therapeutics by enhancing the efficacy of chemotherapy and improving outcomes in breast cancer patients.

## Figures and Tables

**Table 1:** The clinical trials currently deploying CCR5 inhibitors for the treatment of refractory cancers.

NCT	Trial Title	Drug(s)	n	Phase	Status	Results
NCT03274804	Combined PD-1 and CCR5 Inhibition for the Treatment of Refractory Microsatellite Stable mCRC (PICCASSO)	Maraviroc (CCR5 antagonist) + Pembrolizumab (PD-1 inhibitor)	20	I	Completed	Median survival from ~6 mo to >9 mo Median PFS: 2.1 mo (95% CI 1.68–2.30) Median OS: 9.83 mo (95% CI 5.59–20.02)
NCT03838367	Leronlimab (PRO 140) Combined With Carboplatin in Patients With CCR5+ mTNBC	Leronlimab (CCR5 mAb) + Carboplatin	48	Ib/II	Active, not recruiting	Phase 1b: 8/10 patients—stable or regressed 72% decrease in CAML 30 days post tx linked to 300% increase in mean PFS + 450% increase in OS (12 mo)
NCT04504942	Basket Study of Leronlimab (PRO 140) in Patients With CCR5+ Locally Advanced or Metastatic Solid Tumors	Leronlimab (CCR5 mAb)	30	II	Active, not recruiting	Pooled data (n = 19) “>75% improved mPFS 6.1 mo (95%CI 2.3–7.5) & mOS 12+ mo (95%CI 5.5–12+)” Reduced circulating TACs in 75% (n = 21/28) pts (strong predictor of improved survival)
NCT03631407	Safety and Efficacy of Vicriviroc (MK-7690) in Combination With Pembrolizumab (MK-3475) in Participants With Advanced/Metastatic Microsatellite Stable (MSS) Colorectal Cancer (CRC) (MK-7690-046)	Vicriviroc (CCR5 antagonist) + Pembrolizumab (PD-1 inhibitor)	41	II	Completed	Vicriviroc Dose Level DL1: 150 mg (n = 20) DL2: 250 mg (n = 20) mORR DL1: 5% (95% CI 0.1–24.9) DL2: 5% (95% CI 0.1–24.9) mPFS DL1: 4.0 mo (95% CI 2.7–5.6) DL2: 4.9 mo (95% CI 3.1–8.0) OS DL1: 4.6 mo (95% CI 2.7–12.6) DL2: 5.3 mo (95% CI 3.2–8.0) Abort Tx due to AE DL1: 4/20 DL2: 7/20
NCT01736813	CCR5-blockade in Metastatic Colorectal Cancer	Maraviroc (CCR5 antagonist)	12	I	Completed	5/11 pts re-exposed to chemotherapy 3 of those 5: ORR favorable to response rates in pts with mCRC, on or after the third line of chemotherapy, 5–10%. PET-MRI image from 1 pt with advanced-stage mCRC refractory to standard chemotherapy showed clear tumor shrinkage after maraviroc treatment
NCT03767582	Trial of Neoadjuvant and Adjuvant Nivolumab and BMS-813160 With or Without GVAX for Locally Advanced Pancreatic Ductal Adenocarcinomas	BMS-813160 (CCR2/5 dual antagonist) + Nivolumab (PD-1 mAb) +/− GVAX	I: 30	I/II	I: Completed II: Recruiting	Phase I: 9/13 pts proceeded to immunotherapy after neoadjuvant chemotherapy + rad 3 pts received treatment at DL1 6 pts at DL2. No DLTs observed grade 3+ AE: 1 pt
NCT04721301	Ipilimumab, Maraviroc and Nivolumab in Advanced Metastatic Colorectal and Pancreatic Cancer the LUMINESCENCE Trial	Maraviroc (CCR5 antagonist) + Ipilimumab (CTLA-4 mAb) + Nivolumab (PD-1 mAb)		I	Active, not recruiting	
NCT04123379	Neoadjuvant Nivolumab With CCR2/5-inhibitor or Anti-IL-8) for Non-small Cell Lung Cancer (NSCLC) or Hepatocellular Carcinoma (HCC)	BMS-813160 (CCR2/5 dual antagonist) + Nivolumab (PD-1 mAb) + BMS-986253 (IL-8 mAb)		II	Recruiting	
